# Lessons Learnt from HIV and Noncommunicable Disease Healthcare Integration in Sub-Saharan Africa

**DOI:** 10.5334/gh.1370

**Published:** 2024-11-12

**Authors:** Jessica S. van der Mannen, Martin Heine, Samanta T. Lalla-Edward, Dike B. Ojji, Ana O. Mocumbi, Kerstin Klipstein-Grobusch

**Affiliations:** 1 Julius Global Health, Department of Global Public Health and Bioethics, Julius Center for Health Sciences and Primary Care, University Medical Center Utrecht, Utrecht University, Utrecht, The Netherlands; 2 Division of Biology of Disease, Department of Biomedical Sciences, Faculty of Medicine, Utrecht University, Utrecht, the Netherlands; 3 Institute of Sport and Exercise Medicine, Faculty of Medicine and Health Sciences, Stellenbosch University, Cape Town, South Africa; 4 Ezintsha, Faculty of Health Sciences, University of the Witwatersrand, Johannesburg, South Africa; 5 Department of Internal Medicine, Faculty of Clinical Sciences, University of Abuja, Nigeria; 6 Faculdade de Medicina, Universidade Eduardo Mondlane, Maputo, Moçambique; 7 Instituto Nacional de Saúde, Marracuene, Moçambique; 8 Division of Epidemiology and Biostatistics, School of Public Health, Faculty of Health Sciences, University of the Witwatersrand, Johannesburg, South Africa

**Keywords:** integrated care, noncommunicable diseases, human immunodeficiency virus, low- and middle-income countries, Africa

## Abstract

In sub-Saharan Africa (SSA), a rising burden of noncommunicable diseases (NCDs) coexists with a persistent high burden of human immunodeficiency virus (HIV). Integrating care for chronic conditions is potentially beneficial, but the optimal approach remains unclear. By use of a narrative review of 14 recent case studies from different SSA countries, examples of NCD and HIV healthcare integration were described. Case studies were categorized into three models: integrating NCD care into existing HIV care (*n* = 8), integrating HIV care into existing NCD care (*n* = 2), and simultaneous implementation of HIV and NCD services (*n* = 4). Facilitators include staff and patient education, while barriers encompass the lack of guidelines and inadequate infrastructure. Providers, patients, and policymakers support integrated care but note several challenges. Available health economics data suggest cost-effectiveness in the long run. Concluding, NCD and HIV healthcare integration in SSA was deemed feasible with models of service integration related to the implementation context.

## Colliding Epidemics: The Burden of HIV and NCDs

Besides facing a high human immunodeficiency virus (HIV) burden, sub-Saharan Africa (SSA) experiences an exponential increase in the burden of noncommunicable diseases (NCDs) (1). Worldwide, NCDs were estimated to have caused approximately 71% of all deaths in 2016, with 78% occurring in low and middle-income countries (LMIC) (2). In Africa, NCD-related disability-adjusted life-years matched those from communicable, maternal, neonatal, and nutritional conditions in 2017 (3). And it is expected that by 2030, the mortality from NCDs is going to exceed the mortality of communicable diseases (3). Meanwhile, widespread use of highly active anti-retroviral therapy (HAART) has extended the life expectancy of people living with HIV (PLHIV), causing a shift to age-related chronic conditions, such as hypertension and cardiovascular diseases (CVDs) (4), as well as the occurrence of co- and multimorbidity (5). About 25% of PLHIV have hypertension, a major cause of CVD mortality worldwide (4). This has been attributed to the inflammatory effect of HIV infections on vascular walls and the side-effects of several HIV-treatments (6). In addition, PLHIV are (people living with HIV) often pre-disposed to NCD risk factors such as harmful use of alcohol, smoking, poor mental health, unhealthy diet, and physical inactivity (7).

## Integrated Chronic Care: Breaking Down Silos

An integrated healthcare approach, simultaneously delivering NCD- and HIV care as a means of providing the care that meets the individual’s needs has been advocated (8). Both HIV and NCDs require chronic treatment; likewise, they can use similar supply chain management and resources (9). Integrated care in this context involves the management of PLHIV, people with NCDs or those with a combination of both within the same healthcare facility such that patients are treated by the same clinical staff for (all) their condition(s) (10). All patients and providers share the same services within the clinic, such as a waiting room, pharmacy, and tracing system – independent of their disease status (9). However, integration models are still in their infancy with scarce information on the clinical feasibility of healthcare service integration in SSA (11,12,13).

Therefore, this narrative review aims to describe examples of existing models of integrated care in SSA, highlights providers, patients, and policymakers perspectives and discusses challenges and opportunities aimed to guide future integration care programmes.

## Expected Benefits of Integrating HIV and NCD Care

In most SSA countries, vertical programmes in primary and secondary healthcare clinics provide screening and care for a single chronic condition at a time and often refer patients with multiple health conditions to different facilities (14). Increasing numbers of PLHIV with or at risk of NCDs make an integrated one-stop clinic healthcare approach desirable (15,16,17). Potential benefits include that service delivery and management decisions are more coordinated with less duplication and fragmentation of healthcare resources (9,10,11,18,19), as well as a more efficient medication supply mechanism and laboratory capacity, a skilled workforce that offers broader services, and the engagement of communities to, among others, destigmatize HIV (20,21,22). Additionally, there is the potential to reduce costs for both patients and providers by allocating fixed costs, increasing the efficiency of scope and service accessibility, as well as reducing transportation and waiting time for patients (23,24,25). Moreover, the improvement in treatment and adherence of dual-burdened patients might increase the survival rate of PLHIV (16) and reduce the NCD burden (8).

The establishment of successful HIV healthcare programmes in SSA creates a unique opportunity to incorporate NCD care without needing to establish full health service streams (12,20). HIV and NCDs are both chronic diseases where patients need life-long medication and regular check-ups. The existing widespread community structures, workforce, health facilities, drug supply and task shifting could be a suitable starting point for the integration of dual healthcare services (15,19,25).

However, there is also concern that the successes of HIV care are destabilised due to the shift of focus by healthcare providers and subsequent reduced access and quality of care (15,24) and potentially adverse effects on other services (26,27). This could be due to more crowded health facilities with a lack of space and inadequate supplies, a challenging workflow, and the overburdening of providers (11,28). NCD care is often perceived as broad and complicated with low funding support with integrated care models prone to poor governance in resource allocation (15). Additionally, HIV-negative patients might prefer not to attend the same clinic with PLHIV due to stigmatization and therefore drop out of their chronic NCD programme (10).

Eventually, the success of integrated healthcare services hinges on the effectiveness and implementability in diverse settings, its ability to reach the patients and service delivery over time (15). This depends on a wide range of factors that are nationally, regionally or even locally different, such as the existing healthcare system, available resources, and the epidemiology of the chronic conditions (18,21,29). To support the development of appropriate strategies towards increasing healthcare for chronic conditions, it is important to evaluate previous integration programmes in SSA and the lessons learnt from them (30).

## Models of Integrated Chronic Care for HIV and NCDs

Three common models of integration have been identified (9): (1) NCD care integrated into existing HIV care facilities; (2) HIV care integrated into centres originally providing NCD care; and (3) the simultaneous implementation of NCD and HIV care in the same clinic. Several models will be highlighted in this section and, if available, their outcome evaluations and learnt lessons will be discussed. Table 1 provides an overview of case studies with provided services, years of implementation and main findings. ‘These case studies were identified through a systematic search in PubMed using medical subject headings: “Delivery of Health Care, Integrated” and “Noncommunicable Disease” or “Non-infectious Disease” and “Communicable Disease” or “Infectious Disease” and “Africa, South of the Sahara/epidemiology”. In the selection of the cases, we focussed on articles written in English published recently (<5 years) or of particular relevance’.

**Table 1:** Overview of all discussed cases.


STUDY	COUNTRY	PERIOD	SERVICES	NCD CONDITIONS	FACILITATORS	BARRIERS

*Model 1: NCD care integrated into existing HIV care*						

**AMPATH** (9,45,46)	Kenya	2010–2013	– Screening at home, local health facilities and hospitals – Referral of patient – Training of CHW	Diabetes Hypertension	– Improved BP – Reduced glucose levels	– None reported

**Integrated Chronic Diseases Management (ICDM) model** (42,43,44)	South Africa	Since 2011	– Reorganization of facilities – Training of healthcare staff – Providing clinical guidelines – CHW for adherence support and screening – Strengthened supply chains	Asthma COPD Diabetes Epilepsy Hypertension Mental health	– Increased BP control – Increased HIV control	– Inadequate adaption of care model by staff – Underresourced facilities – Lack of privacy – Lack of patient education

**Integrated Chronic Care Clinics (IC**3**)**(12,36)	Malawi	2014–2015	– Screening in the clinic – Patient education – Free supply of medication – Referral of patients – CHW for adherence and social support – Training existing staff, including reducing high turnover rates – Patient masterfile	Asthma Diabetes Epilepsy Hypertension Mental health	– Increased NCD service accessibility – Improved clinical outcomes and retention rates – Stable HIV control	– None reported

**TASSH intervention** (30,39)	Nigeria	Since 2014	– Screening at primary health centres – Lifestyle counselling – Referral of patients – Taskshifting by nurses	Hypertension	– Reduced BP – Increased HT control – Reduced HIV stigma	– Lack of medication – Insufficient referral system – Lack of policies

**HIV Clinic at Zomba Central Hospital** (16,25)	Malawi	2016–2017	– Taskshifting by existing staff – Patient counselling – Screening at hospital – Using expert clients for data collection – Provision of medication – Electronic medical patient file	Diabetes Hypertension	– Saving time for patient and provider – Improving awareness among patients	– Increased workload – Shortages of tools

**NAMPROPA (QIC)** (41)	Namibia	2016–2018	– NCD screening at HIV facilities – Training of nurses for counselling and referrals – Health volunteers for data collection	Hypertension	– Increased number of NCD cases	– Low linkage to care rate

**SEARCH Study** (22,28,29,30,31,32,33,34,35,66)	Uganda Kenya	2018–2020	– Health campaigns in communities, offering screening and treatment. – Training existing healthcare staff – Providing a pleasant clinical environment – Patient education	Hypertension	– Increased HT control – Stable HIV control – Lower mortality	– Medication stockouts

**Mulago clinic** (37,38)	Uganda	2019–2020	– Routine screening in clinic/hospital – Delivery of care – Patient education – Taskshifting by nurses – Free supply of medication	Hypertension	– Increased HT control – Stable HIV control	– Lack of medication – Lack of policies – Lack of patient documentation

*Model 2: HIV care integrated into NCD care*						

**CIDRZ programme** (9)	Zambia	2008–2011	– HIV testing and counselling at primary healthcare facilities – Enrolment in same facility	Outpatient clinic	– Increased number of HIV case findings – Increased efficiency of space, resources and staff time – Decreased HIV stigma	– None reported.

**Government Reproductive Child Health Clinics** (9)	Tanzania	2010–2013	– HIV screening for cervical cancer patients – Referral of patients	Cervical cancer	– None reported.	– Lack of enough equipment

*Model 3: Simultaneous implementation of NCD and HIV care*						

**Collaborative programme** (9)	Uganda	2011	– Screening in communities – Referral of patients – Patient education – Arrangement of transportation	Diabetes Hypertension	– Increased number of HIV and NCD case findings	– Low linkage to care rate

**Ministry of Health** (9)	Lesotho	2011	– Screening in communities – Referral of patients	Diabetes Hypertension	– None reported.	– Low linkage to care rate

**Homebased testing (Linkages Study)** (23,28,51)	South Africa	2012–2013	– Screening at home – Health counselling – Linkage to care nearby – Training of CHW – NCD risk factor screening	Cholesterol Diabetes Hypertension Mental health	– Increased number of HIV and NCD case findings	– None reported

**MOCCA (INTEAFRICA)** (10,11,19,48,49,50,65)	Tanzania Uganda	2018–2020	– Screening and treatment at clinics – Training of existing staff – Health counselling	Diabetes Hypertension	– Improved retention rates – Stable HIV control – Acceptability by patients and providers	– Long waiting time – Lack of medication – Fear of HIV stigma


Includes a summary of the main services provided by the programme, the years of performance and a summary of the main findings per case. BP = blood pressure; COPD = chronic obstructive pulmonary disease; CWH = community health worker; HIV = human immunodeficiency virus; HT = hypertension; NCD = non-communicable disease.

## Case Studies Under Model 1: NCD Care is Integrated in Existing HIV Care

The NCD to HIV model appeared the most applied model of integration in literature (5,31,32). This integration model relies on the already existing network of government policies, supply chain networks and healthcare facilities that have previously been established during the 90–90–90 UNAIDS goals (9). The long-term care that is necessary to manage chronic NCDs can benefit from the infrastructure of long-term HIV care, including medication provision, clinical monitoring, and patient tracing (22). Patients can be enrolled in this model if they have either one or multiple diagnoses, and they could be referred from other clinics, respectively through screening or case-finding activities in the community (9). The reported requirements for the implementation of this model are: (1) providing training to medical staff to perform both HIV and NCD screening, diagnosis, and chronic treatment; (2) effective medical record keeping, including patient tracking systems to sustain adherence to treatment; (3) having an appropriate supply chain network for the provision of equipment and medication; and (4) providing health education to patients.

This model of integration is for example represented in the SEARCH study in rural Uganda and Kenya where 32 rural communities participated in a community-based universal test-and-treat strategy for HIV and NCDs (22,33,34,35). Each community organized a community health campaign where residents were screened and if necessary, directly linked to care at a nearby health centre. This multi-disease streamlined model of care includes the training of clinical staff to provide a welcoming environment, reduce waiting times by promoting clinical transit, and have an overall improved patient-centred care system. Patients only had to access the clinic occasionally to receive treatment for all their conditions simultaneously, and a telephone hotline was available for any additional information. In addition, patients received tips on lifestyle adaptation to improve their health. After successful implementation of the SEARCH approach, hypertension control increased from 15% pre-implementation to 46% post-implementation (22) while keeping HIV viral load suppressed; mortality rates were reduced by 23% in the integrated care clinics (33). However, higher hypertension control was limited by regular medication stockouts and too frequent medical appointments which discouraged patients (22).

NCD care was also integrated in screening, health education and free provision of medication offered to patients at fourteen Integrated Chronic Care Clinics (IC3) in Malawi (36). Both PLHIV and/or patients with NCDs were referred to the closest IC3 from other clinics or through community screening events, using the original patient flow established in the HIV model of care (12). Overcrowded clinics and overburdening of healthcare staff were prevented by introducing an efficient patient flow system by treating the most ill patients first and providing monthly (rather than 3-monthly) check-ups for the more complex diagnosed patients. The IC3 programme relied on a vast network of community health workers to assist patients with adherence and social support, and they would perform patient tracing in case of a missed appointment. Existing healthcare staff would be trained in managing both HIV and NCD patients, and additional hospital staff would travel to nearby facilities a few times per week to reduce IC3 staff shortage. An important part of the strategy was to retain the same clinical staff available at the clinic to ensure long-term investment and the transformation of regular healthcare providers to integrated chronic care providers. Patient data were filed in a master file which included information on appointments, conditions and medication, which improved monitoring and programme progress (12). In this programme, an increase in NCD service accessibility through the maximisation of clinic staff, space, and resources and overall improved clinical outcomes and retention for patients was seen, without interfering with HIV outcomes (36).

At a clinic in Mulago, Uganda, HIV patients are routinely screened for hypertension (37,38). This clinic used the WHO HEARTS strategy to deliver hypertension care to its patients. This strategy consists of six components (38): 1) health education, including medication adherence, and lifestyle counselling for patients; 2) routine screening during each visit; 3) task shifting by nurses to prescribe hypertension medication; 4) implementing evidence-based hypertension treatment protocols; 5) consistent supply of hypertension medicines for free; and 6) hypertension-specific monitoring and evaluation tools. When a patient was screened positive for HIV and hypertension, he/she would receive medication for both diseases from the same clinician and have future check-ups for both conditions simultaneously (37). Additionally, the clinic offered a 5-day campaign in its community, where it identified a high burden of undiagnosed NCDs. The study showed an increase in hypertension control among patients from 9% pre-implementation to 74% 6-months post-implementation (38). Moreover, the quality of HIV care was maintained as 99% of the patients sustained controlled pre- and post-implementation. Several facilitators and barriers were experienced in this integrated care model (37). Facilitators include leveraging HIV treatment adherence support to NCD treatment adherence support by peer educators, healthcare providers having adequate knowledge and skills to provide screening, having enough measurement equipment, and being able to receive care at only one clinic. Barriers include lack of patient education and provider treatment knowledge, absence of monitoring and adequate treatment protocols, delayed delivery or lack of medication and its high costs, inadequate equipment maintenance, lack of documentation leading to frequent changes in medication, failed task-shifting actions from nurses, and finally patient’s prioritization of HIV treatment adherence over NCD treatment adherence.

The Task-Strengthening Strategy for Hypertension Control (TASSH) study in 32 community-based primary healthcare centres in Nigeria aims to improve the accessibility of essential services through efficient use of healthcare and community health workers and the ability for patients to access care in one facility (30,39). The TASSH intervention implementation consists of three phases: (1) development of a tailored facility intervention to improve TASSH into the HIV clinic; (2) intervention implementation and evaluation of the clinical effectiveness; and (3) post-implementation evaluations of the adaptation and sustainability of the TASSH intervention in the facilities. Participating facilities provide CVD risk assessment, medication titration, lifestyle counselling and referral for all PLHIV who are diagnosed with or at risk of CVD. For each participating facility, a context-specific approach was developed to implement TASSH into the facility as part of phase 1. For this, a steering committee with key stakeholders was set up to provide leadership and experienced nurses were capacitated to train the participating nurses to perform CVD risk assessments, prescribe medication and refer patients. Facilities that implemented TASSH showed a significant reduction in systolic blood pressure and an increase in hypertension control. This is in line with findings from a previous TASSH intervention study undertaken in Ghana (40). Facilitators that contributed to this success were: (1) reduced workload for healthcare staff; (2) improved care efficiency; (3) access to care in a one-stop clinic for patients; (4) reduced waiting times and HIV stigma; (5) improved referral systems; (6) health education for NCD management; and (7) supporting patient’s need and providing professional incentives. Identified barriers included: (1) potential disagreements over role and role boundaries by healthcare staff; (2) lack of equipment and medication; (3) not having a sufficient referral system and tracing system; and (4) lack of national policy implementation in the clinic.

Implementation of an integrated care approach in an HIV Clinic at Zomba, Malawi included training of health workers to provide task-shifting services of integrated screening and care to patients (25). Expert clients were employed to perform data collection, patient counselling, and screening for hypertension and diabetes. Patients were prescribed and provided with medication directly, when available. Additional lifestyle advice was given to improve their overall health. During the initial implementation of the integrated services, the average appointment time increased. However, once implemented, appointment time returned back to normal, suggesting the provision of integrated services being feasible without increasing appointment time (25). Overall, a high uptake of integrated screening in the clinic was observed. Lessons from this integrated care intervention include: organising an efficient patient flow, implementing useful monitoring tools, and using an electronic medical patient file system (25). Interviewed healthcare providers considered this intervention to save time for both staff and patients, reduce hospital visits, and improve awareness of health status among patients (16). Negative aspects included shortages of measurement tools, increased workload, and a lack of interest by HIV health staff to spend time on NCD care.

The NAMPROPA (QIC) project provided free healthcare services to improve NCD screening and treatment into routine HIV care in 24 HIV facilities at three high-burden HIV regions in Namibia (41). The Ministry of Health-funded program involved NCD screening during medication pick-up at HIV clinics and subsequent NCD screening during every first patient appointment. Nurses were initially trained by health mentors to provide NCD counselling and referral, and health volunteers were trained by nurses to record NCD data in health registers. As for most SSA countries, nurses are allowed to perform screening and prescribe HIV medication, but only clinicians can prescribe NCD medication affecting treatment initiation delays. Electronic health systems were used for all the HIV-patient data, but paper-based patient passports were used to keep track of NCD care and remind patients of follow-up appointments, hindering the supply chain management and continuity of care. While many new NCD case findings among HIV-positive patients were reported, less than two-thirds of them received treatment (41). This has been attributed to the challenges with documentation and execution of referrals, the problem of patients travelling to another facility, long waiting times, and stock-outs of treatment.

In South Africa, chronic NCD care was integrated in HIV care in the Integrated Chronic Diseases Management (ICDM) model. This model consists of four major sections through which the delivery of integrated care to its patients would be optimized (42,43). Firstly, facilities were reorganized to improve patient flow, operational efficiency, and patient satisfaction while reducing waiting times. Secondly, quality of care was reassured through adequate training of healthcare staff, mentoring them, and providing appropriate clinical guidelines. Thirdly, the ICDM model promotes the self-management of patients via community healthcare workers, who provide adherence support, first-line screening, and point-of-care testing in local communities. Lastly, supply chains were strengthened through collaboration with various (communal) stakeholders. Several barriers, mentioned by healthcare staff and patients in integrated care clinics were reported: under-resourced facilities, lack of appropriate training and guidance, lack of standardisation in guideline adoption, lack of privacy, burden of taking multiple medications, lack of patient education, and the costs of treatment. Successful model implementation was hindered by the inability of healthcare staff to adapt to the model and the lack of adequate management for programme implementation. Similarly, patients mentioned the lack of education and support. An effectiveness study of this model showed higher HIV control in ICDM clinics, possibly caused by reduced HIV stigma (44).

In the Academic Model of Providing Access to Healthcare (AMPATH) study in Kenya, all residents of twenty communities participated in HIV and NCD screening at multiple sites: home-based, local health stations, and connected hospitals (9,45,46). This programme uses task-shifting and community partnership models to provide integrated care. Training of community volunteers and successful referral systems are key to the programme. The study showed improved hypertension control, blood pressure, and reduced average blood glucose among participating patients, whilst also improving adherence to HIV care, suggesting positive programme effectiveness.

A recent systematic review summarized the level of cervical cancer care integration into existing HIV healthcare services in SSA (47). Uptake of screening was high and overall, there was a consensus that integrated health care was feasible, safe, and acceptable for both providers and patients. Several studies reported an increase in retention at integrated care facilities via tracing systems, however, the majority still noted a high loss to follow-up for treatment. Facilitators promoting health integration included: (1) the use of pre-existing healthcare infrastructure; (2) healthcare providers’ willingness to collaborate, perform task-shifting activities and have adequate training (train the trainer model); (3) continuous education and/or supervision; (4) having a single-visit approach; (5) implementation of screening protocols; (6) development of a (bi-directional) referral system; (7) having an electronic medical record system and a phone-based tracing system; (8) engagement of stakeholders, including community and peer educators; (9) having adequate resources; and (10) low (transport) costs for patients. Barriers mentioned in this study are: (1) not having enough (educated) staff, staff being overwhelmed and having high staff turnover; (2) loss of patient follow-ups; (3) inconsistent supplies of resources; (4) lack of electronic medical records; (5) long waiting times, delays in access to treatment, and not having enough space; (6) lack of follow-up systems; (7) lack of financial incentives to providers and having inconsistent quality between providers; (8) high costs and long waiting times for patients visiting the clinic and receiving treatment; and (9) fear of diagnosis, test results and treatments for patients.

## Case Studies Under Model 2: HIV Care Integrated into Centres Originally Providing NCD Care

This second model focuses on the integration of HIV services into a healthcare facility that is already providing NCD care and arguably is the rarest model of integration (9,32). Patients enrolled in these facilities were offered HIV testing and, if necessary, treatment at a nearby HIV clinic or within the same facility. The requirements for the implementation of this model are (1) training of clinical staff to perform HIV counselling, testing and care; (2) the development of efficient referral tools; (3) health education for patients; and (4) adaption of medical records and supply chain to provide screening and, if possible, treatment.

Twenty primary healthcare facilities in Zambia participated in the integrated care programme of the Centre for Infectious Disease Research in Zambia (CIDRZ) (9). Patients attending one of the facilities were offered HIV testing and counselling during regular appointments (9). An HIV-positive patient would be enrolled into HIV care within the same healthcare facility. In this way, patients could receive simultaneous care for all their conditions. Successful integration of HIV services into primary healthcare facilities has been reported, with an increased efficiency of clinic space, resources, and staff time (9). Additionally, they reported an increase in HIV case findings and a decrease in HIV stigma.

The Government Reproductive Child Health Clinics in Tanzania fall under model 2, however, are only limited to patients with cervical cancer (9). HIV is a risk factor for women to develop cervical cancer, therefore, is it not unlikely to find women unknown of their HIV status while being treated for cervical cancer. An opportunity for healthcare providers to detect additional cases. Patients who test positive for HIV are subsequently referred to an HIV clinic nearby. However, testing equipment was not always available, limiting the continuity of this intervention.

In the Médecins Sans Frontières programme in Kenya, two primary health facilities were offering NCD and HIV screening and treatment (9). There was an increased number of patients who adhered to their programme at these facilities compared to fragmented care clinics.

## Case Studies Under Model 3: Simultaneous Provision of HIV and NCD Care

In this last model, screening for multiple conditions is offered simultaneously in either existing healthcare facilities or through community outreach services (9). Patients that are tested positive for HIV are subsequently referred to a nearby non-integrated clinic. The requirements for the implementation of this model are (1) training of healthcare providers and community workers to provide community screening; (2) patient education; and (3) efficient referral protocols.

In the MOCCA (INTE-AFRICA) project in Tanzania and Uganda, ten existing healthcare facilities, ranging from small clinics to regional hospitals started with the provision of integrated care (10,11,19,48,49,50). These facilities previously provided NCD and HIV care on different days, and sometimes in different buildings. Under the programme, NCD and HIV care was offered simultaneously on one or two designated days in the same building. At the integrated care clinics, all patients share the same facilities, healthcare staff, patient record systems, tracing system, and pharmacy. Before the start of the programme, all necessary equipment was provided, and all participating staff received training on counselling for and managing NCD and HIV. Additionally, there was a small medication supply buffer installed to take in any interruptions in supply. After some start-up hiccups, the programme showed a great retention rate, without affecting HIV control (10). Improved adherence was attributed to patients following their doctor’s advice, being better informed, and saving time on travelling to only one facility (49). The overall acceptability of integrated care by both healthcare providers and patients contributed to the success of the MOCCA trial. Concerns were related to transportation costs, waiting times, and lack of specific diabetes and hypertension medication (50). Furthermore, non-PLHIV were afraid to be perceived as HIV-positive when being treated in the same facility, resulting in reduced acceptability of integration. Additional efforts are therefore needed to address privacy and confidentiality for all patients. The integrated model for delivering care to individuals with HIV, diabetes, and hypertension was shown to enhance the availability of diabetic and hypertensive care while maintaining the quality of HIV care (50).

In KwaZulu-Natal, South Africa, as part of the Linkages Study, residents were offered home-based screening for HIV and NCDs (23,51). Patients would receive additional counselling and linkage to care at nearby facilities. To have enough staff, nurses trained community volunteers to conduct NCD and HIV screenings, perform anthropometrical measurements and complete interviews about health and lifestyle with patients. Even though a high prevalence of HIV and NCD-positive patients was reported, no information was provided about the number of patients receiving care (51). As part of the additional workload for healthcare staff, 15%–20% fewer clients could be attended to as compared to pre-implementation (28). It was recommended to use a more efficient screening protocol to decrease time spent per patient to address this barrier.

In a similar case study providing NCD and HIV screening in three community centres in Uganda, patients were referred to existing clinics as the temporary community screening centres did not provide any treatment yet (9). The programme observed a significant increase in HIV- and/or NCD-positive patients to whom they provided education on their condition and arranged transportation so they could visit other clinics. Slightly less than half of the positive patients were receiving treatment.

Similarly, mobile service delivery points in rural Lesotho communities provided NCD and HIV screening but no treatment to their residents (9). Patients were referred to non-integrated healthcare facilities. Less than one-quarter of the patients that were tested positive for a condition during this campaign ended up receiving treatment.

## Factors Facilitating Integration of NCD and HIV Care

**Figure 1:**
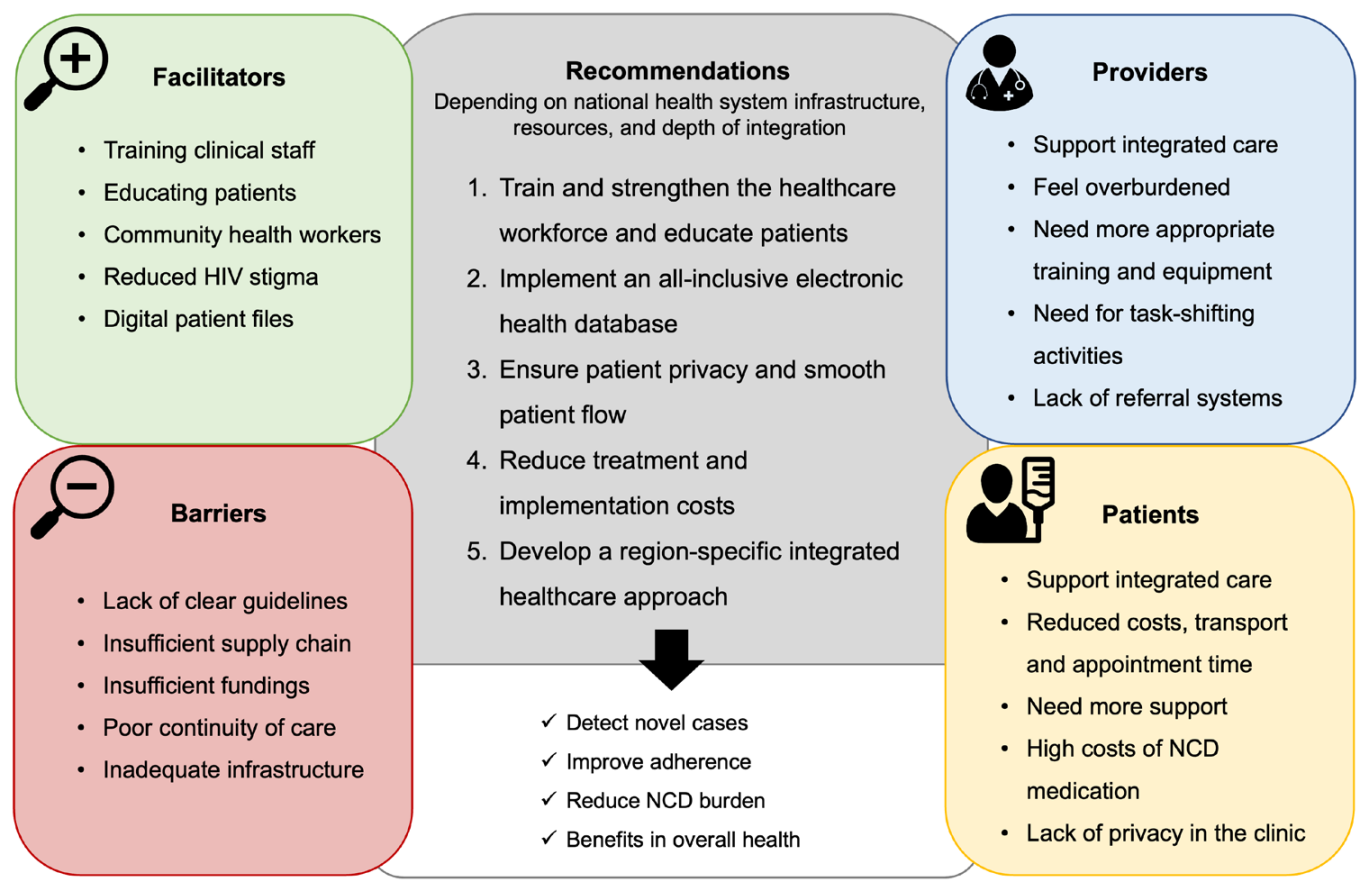
**Lessons learnt from HIV and noncommunicable disease healthcare integration in sub-Saharan Africa**. Graphical overview of the narrative review with the main findings. Several facilitators and barriers, combined with the perspective of providers, patients, and policymakers resulted in the recommendation for the development of future healthcare integration programmes in sub-Saharan Africa (SSA). The goal of successful integrated health is to detect novel cases and improve patient adherence to reduce the NCD burden and overall health in SSA. HIV = human immunodeficiency virus; NCD = noncommunicable disease.

Figure 1 provides a graphical synthesis of the barriers, facilitators, benefits, and lessons learnt for integrated NCD and HIV care. Integration models that only provided screening had the advantage of detecting novel cases and providing a platform for patient education, improving lifestyle choices and health. Models that also provided treatment added the benefits of consistent health monitoring, the management of multiple needs for patients, and the improvement of health status. These positive outcomes were reported in a systematic review and meta-analysis on integrated care, observing a significantly higher treatment success for NCDs and uptake of NCD services in integrated programmes as compared to non-integrated NCD programmes (18). Strategies reported to facilitate integrated care included free-of-charge services, clear policies, using existing healthcare providers and local leaders to support the acceptability of implementation, providing continuous medical supervision and treatment medication, and having patient feedback (32,47).

A literature review researched the role of communication change to promote NCD and HIV interventions in SSA (15). Health education interventions, motivational interviews, and peer group support at a community level were reported to promote healthy lifestyle decisions, resulting in a lower disease burden (15). In addition, mobile health interventions were observed to be useful in the improvement of healthcare access in rural areas by providing online training to health providers, and screening and diagnosis to patients.

A simulation model based on HIV/NCD dynamics in a national and regional setting assessed the epidemiological impact of the SEARCH programme in Kenya (29). The implementation of an integrated model resulted in a 64% reduction in HIV prevalence, and around a 30% reduction in NCD prevalence (including hypertension, diabetes, and CVDs).

## Barriers limiting Integration of NCD and HIV Care

Barriers encountered during the implementation and execution of integrated models of care can be allocated to the different levels of healthcare: system, providers, and patients.

On a system level, barriers included: overburdened health facilities, partial integration in the continuum of care, lack of linkage to care after referral, lack of logistics and management, high NCD care costs, insufficient resources, lack of functioning equipment, challenges in medication supply, and limited access to amenities, protocols, and guidelines (8,9,12,14,24,26,28,32,41,47,52,53,54).

On a provider’s level, the lack of NCD training and knowledge, poor continuity of care in facilities that do not provide treatment, inadequate medication prescription and counselling, having an understaffed team, providing low quality of care, and not receiving enough recognition and incentives, were noted (18,24,26,28,37,38,52,53,55).

On a patient level, the lack of patient education resulted in the lack of knowledge and awareness among patients, which has been associated with patients feeling stressed, depressed, and overwhelmed by their health conditions and more likely to stop taking their medication (56). In addition, the lack of patient’s ability to take some time off work to visit the clinic during weekdays due to commitment to work, and sometimes asymptomatic nature of NCDs, has been observed to be one of the reasons for lower levels of adherence to clinic visits and medication (12,24,32,37,47,48,49,53). Additionally, long waiting times, have been mentioned as reasons impeding clinic visits (49,57).

The system-related barriers were the most dominant and caused barriers on patient levels (52). Low prioritization of NCD care on a national level has been shown to result in a lack of management skills and, subsequently, inadequate NCD management and training, translating into poor counselling, medication prescription, and adherence (52). As NCDs are less likely to be prioritized, NCD care suffers from a lack of attention, prioritization, and funding (24). Additionally, clinics with a high staff turnover are less likely to have successful integration as training new medical staff requires a lot of funding. Inadequate staff training and a lack of coordination resulted in a lower HIV and/or NCD uptake in the integrated care clinics (18).

## Provider Perspective

Providers play an important role in the implementation of integration models and their success. They have to execute the policies and guidelines of health programmes. Additionally, their understanding and attitude towards care reflect on the quality of care and on the acceptability among patients. It is therefore important to report on the providers’ perspective on healthcare integration.

Overall, healthcare staff has a consensus about the benefits and need for integrated care as the treatment of multiple chronic conditions share common elements that can enhance incorporation. But they also address the need for appropriate training, coordination, and management (8,24,39,52). Training is time and resource-intensive especially at the beginning of programme implementation and at facilities where there is a high staff turnover (9). Additionally, healthcare workers unanimously agree on the increased workload that comes with integration and the problem of staff shortage. This makes some of them prioritize conditions that they perceive as being more important (16,24).

Healthcare providers also noted the lack of appropriate tools, such as working machines, adequate documentation, up-to-date health guidelines, and protocols (37,52,53). Other limitations such as lack of medication and laboratory facilities were equally mentioned (16,52). This often resulted in the referral of patients to other facilities which burdens the patients and since the referral networks were not always efficient, patients were left without adequate treatment (39).

Within the healthcare setting, a noticeable gap exists between doctors and nurses. Nurses are permitted to conduct screenings, yet initiating treatments is only reserved for doctors (37). This not only leaves nurses feeling less engaged but also introduces delays in the onset of treatments. It is this very gap that could probably be a future solution to the overburdening of healthcare staff, offering a more efficient utilization of the existing workforce through task shifting. (17,39,58). The differences in national health systems and laws between countries on task-shifting strategies mandate the possibility of such implementation. Moreover, leveraging community health workers could further improve care as they can perform primary screening and support, and counsel patients to improve lifestyle and adherence (30,37,49,52).

Healthcare providers gave several other suggestions on how to improve the implementation of integrated chronic care (52,59). Among these are the need for equal disease prioritization and education, adequate financing, and improved resource allocation, but also capacity development and enhanced infrastructure to provide for all patients. Additionally, the provision of digital services to improve provider-patient contact and increase retention rates, the installation of monitoring indicators, performance targets, and adequate systems for data collection could, according to providers, improve health services (37,52). Furthermore, they suggest the need for incentives in the form of professional development and social recognition (39).

## Patient Perspective

The patient’s experience and attitude towards care are important for health outcomes and the burden of disease. Overall, patients were satisfied with integrated care as a more holistic treatment approach because it reduced transportation costs and appointment time, as well as household income reduction due to disease since less time of work had to be taken for care, and transportation costs were lower (60). These factors contributed to improved retention rates and health status among patients attending integrated clinics (16,37,49,61). Concerns were related to longer consultations and longer waiting times in the case of attending to multiple health conditions (19,61). Therefore, there is a need to inform the public on the benefits of integrated care, educate patients on HIV and NCDs and their risk factors to improve retention rates and ability to self-manage (8,9,60). The use of peer educators would increase the acceptability among patients as they are regularly more trusted (37,61).

Patients described not taking NCD care seriously because of regular medication stock-outs and the lack of continuity of care because of high staff turnover rates (24,53,56). Another important point patients raised is the need for enhanced confidential practices (53,56). In some clinics, the physical shortage of available space made it difficult to communicate with only one patient at a time and this loss of privacy made patients less likely to return to the clinic (52). As sometimes medical records were not considered private, HIV-negative patients feared being perceived as HIV-positive by their peers because of the non-segregated contact (49). On the contrary, reduced HIV stigma has also been mentioned by several different integration studies (19,53,56,60). And despite the lack of space, improved provider–patient relationship was reported with the integrated care approach as clinicians were aware of the full medical record to provide adequate counselling (49).

Finally, the costs of NCD medication were also an important factor among patients to discontinue their treatment (37). HIV medication is provided for free, removing a financial barrier to patient adherence, however, patients often have to pay for their own NCD medication. Furthermore, as some clinics made patients come back frequently for check-ups, there is an additional financial burden on patients as they have to take time off work and pay extra for transportation (8,52). Clinics that only provided screening, but no treatment made referrals to other facilities, but this was not always possible for patients and was generally associated with additional costs (53). Overall costs for patients attending all levels of service at an integrated care facility were shown to be lower on an annual basis than those receiving fragmented services (56).

## Policymaker Perspective

Policymakers carry the responsibility to design the strategies and give guidelines through which NCD and HIV healthcare services should be integrated. Their vision on healthcare integration will resonate to the development, prioritisation, and eventually, the content of the policies and training that providers have to follow, and the care patients will receive (62,63). Overall, integration is viewed positively in relation to health systems and patient benefits (19,64). The current lack of evidence-based integration models makes it difficult to develop effective and contextually appropriate policy and practice-based strategies (13,17,41).

During the evaluation of the implementation of the MOCCA trial, policymakers were interviewed on the benefits and pitfalls of the program (19). In this study, policymakers recognised the opportunity to detect undiagnosed conditions by providing more comprehensive screening, early treatment, and care within an integrated setting (65). This approach not only facilitates early diagnosis but also ensures timely and effective treatment. Moreover, the integration of NCD and HIV care also addresses the issue of inadequate treatment for NCDs, especially related to the availability of medicines (19). The financial burden on patients to privately purchase these medicines led to challenges in managing both conditions simultaneously.

Policymakers raised another important benefit of integrated care. When patients visit the same clinic for all conditions, they receive the same health education and awareness. This shared knowledge and experiences among patients with different conditions increases the feeling of understanding and community support, improving general health status (19).

Policymakers also take a broader perspective on the capacity-building of healthcare workers within the integrated care model (63). The integration of HIV care alongside NCD enhances healthcare workers’ knowledge across different conditions and informs the wider research and policy context (19). Policymakers recognise the opportunity to leverage the vast experience and knowledge developed in SSA countries through research and policy in the HIV platform, as described under model 1. This experience, but also the experience of healthcare workers from NCD settings, provides a foundation for developing the rightful framework for the management of HIV and NCDs within integrated settings (19).

However, the efficacy of targeted screening and the cost-effectiveness per SSA country should individually be assessed, together with the national level of health system infrastructure (64,65). Additionally, stakeholders should agree on the desired depth of integration. This can vary from linkage (referrals and sharing of information) to coordination (common policies to address related health issues) and integration (merging of two programmes) (64). Based on these important aspects, policymakers can develop guidelines to improve nationwide integration implementation.

## Cost-effectiveness

Integrated care models may benefit from decreasing costs by sharing fixed costs over all patients and increasing economies of scale (28,45). In addition, the integrated care models might increase the efficiency of scope as they offer a wider variety of disease screenings and treatments that need complementary services (23,28).

Cost-analysis studies of the SEARCH programme showed additional costs for integration were calculated to be only 2.4%–4.0% [hypertension (66)] or 6% [hypertension and diabetes (28)] mainly attributed to treatment medication costs (66). While programme integration may be expensive at first, it is expected that costs decrease with time (28). As shown in the modelling study of the SEARCH programme, this intervention was 90% likely to be cost-effective (29). However, substantive start-up costs are estimated to increase the national health budget by 12%. Therefore, the simulation model suggested starting with a more targeted approach for integration programme implementation in regions with higher HIV prevalence shown to result in the prediction of CVD risk reduction of 19.6% over 10 years when NCD care would be integrated into existing HIV clinics (6). Further reducing the NCD treatment price, should also increase the cost-effectiveness (29). The MOCCA trail showed a one-third to almost a half reduction in healthcare and household costs when compared to a vertical care model (48). Savings were considered to be the result of reduced time spent on appointments for healthcare providers and patients.

However, other studies have shown that integrated care results in a modest increase in costs (23). Most important drivers for the increase of costs were the price of the treatment itself, costs for additional tools, and extra time spent by healthcare providers providing for more than one morbidity in a clinic (28). This was shown in a cost-analysis study of the Linkages study, where the 20–40% increase in healthcare costs was attributed to the costs for measuring tools and staff costs for the provision of additional services (23). However, the use of different, cheaper measuring tools was recommended to weigh up the additional cost with respect to the potential benefit of preventing NCDs.

Cost and cost-effectiveness study stakeholder interviews from different countries highlighted several challenges with the integration of NCD and HIV care (28), including current funding streams, lack of adequate infrastructure and equipment, medication allocation, medication (NCD) costs, availability of treatment protocols, and appropriate monitoring and evaluation systems (9,24,52).

Hence, at first, it might be more cost-effective to implement integrated care models in regions with high HIV prevalence, as it would be the most efficient way to share resources and reduce costs (29). It is recommended that every policymaker evaluates their own population needs and resource availability to develop an optimal cost-effective programme (28,31).

## Future Perspective

In summary, many of the included case studies indicate that integrated health models improve patient health (6,8,10,12,15,17,18,22,25,29,33,36,41,47,48,49,56,58). They predominantly focussed on the integration of screening practices for hypertension and diabetes into existing HIV care. They observed improved service uptake, testing and counselling, treatment initiation and retention, and improved staff and patient satisfaction. Benefits of integration management are found in the similar characteristics of both target populations that require management for chronic diseases. This facilitates the sharing of resources, staff, and networks and potentially results in lower additional health service costs (19,48). However, successfully implementing an integrated model of care requires intersectoral efforts, including infrastructure, and the lack of evidence-based care models and cost-effectiveness studies makes it difficult to develop suitable policies (17,18,28,67). Considerations for the development and successful implementation of future integrated care models in SSA are highlighted in the grey box in Figure 1 and include:

- Train the healthcare workforce and strengthen it by using task-shifting strategies and the use of community health workers. This helps prevent overburdening of staff and high turnover rates while improving patient education and retention (5,8,9,13,17,19,24,25,36,37,39,41,47,52,55,58,59,63,68).
- Implement an (electronic) health database with a complete overview of a patient’s health status and appointments that can also be used for patient monitoring, tracing, and referrals (5,9,15,18,25,39,52,68,69).
- Ensure patient privacy in clinics and smooth patient flow, this includes less frequent appointments for stable patients or fewer test screening for low-risk patients and the referral of complex cases (12,16,22,24,25,36,48,49).
- Reduce treatment and implementation costs by establishing appropriate funding streams and a continuous supply chain for equipment and medication, and introduce integrated care gradually, starting with high-risk regions and patients (5,6,9,12,13,17,24,25,28,36,37,39,41,47,49,52,59,66,68,69).
- Perform region-specific analysis, meet with stakeholders, and establish a research partnership to ensure a long-lasting implementable and detailed policy for a suitable integrated health model (5,8,13,23,24,26,28,31,39,41,59,64,65,68,69).

From a provider’s perspective, integrating NCD and HIV care could have great benefits for the healthcare system and the patient’s health status. Although integration requires extra work and time, the burden of the additional tasks could be reduced through appropriate training and extension of the workforce. From a patient’s perspective, the advantage of only having to travel to one clinic and possibly only once a month, reduced HIV stigma, and adequate counselling contributes to integration acceptability, improved adherence, and better health. Improving patient education including lifestyle counselling and the importance of lifelong treatment adherence may facilitate better health outcomes.

In conclusion, the integration of HIV and NCD services is considered feasible in SSA countries. However, given different healthcare systems and resources, the development of an integration model requires comprehensive consideration of the provider and patient perspectives and previous programme experiences by the policymakers. The success of integration hinges on the linkage of patients to care. Consequently, well-designed, and well-implemented health integration models could have the potential to divert the NCD burden and improve the overall health in SSA.
